# Geographical Distribution of COVID-19 Cases and Deaths Worldwide

**DOI:** 10.34172/jrhs.2020.24

**Published:** 2020-09-30

**Authors:** Jalal Poorolajal

**Affiliations:** ^1^Research Center for Health Sciences, School of Public Health, Hamadan University of Medical Sciences, Hamadan, Iran; ^2^Department of Epidemiology, School of Public Health, Hamadan University of Medical Sciences, Hamadan, Iran

COVID-19, the disease caused by the new coronavirus, is a highly contiguous infection that spread immediately across the world since its beginning in Wuhan, China on Dec 31, 2019^1^. By Aug 23, 2020, over 23.5 million people were infected and more than 800,000 people died from the disease^2^. Besides several well-known and much deadlier diseases worldwide^3^, COVID-19 is an emerging infectious disease that has caused huge health and economic disaster worldwide.

**Figure 1:**
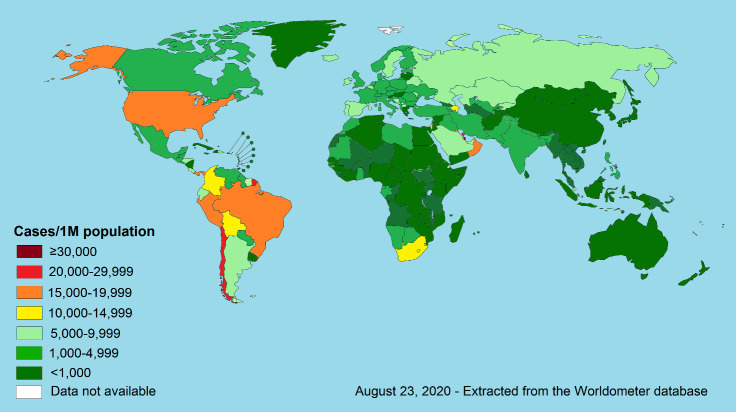
The geographical distribution of the COVID-19 cases per million worldwide

**Figure 2:**
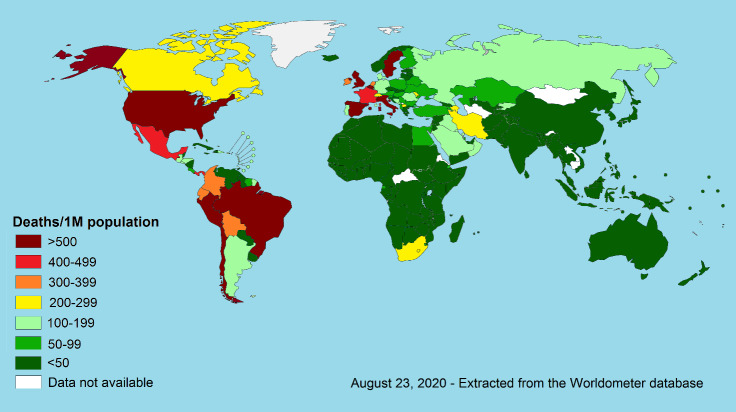
The geographical distribution of the COVID-19 deaths per million worldwide

COVID-19 disease involved almost all countries around the world. Like any other disease, genetic variations in population across different areas of the world may affect the COVID-19 related morbidity and mortality. Moreover, the disease surveillance system and completeness and timeliness of cases and/or deaths reports can cause differences in disease mortality and morbidity rates in different parts of the world. However, the geographical variation in COVID-19 cases and deaths is very wide following a mysterious pattern. As shown in Figure 1, while the prevalence of this disease is very high in the western hemisphere, the prevalence of the disease is not very high in the eastern hemisphere irrespective of the economic levels of the countries. A majority of countries with a prevalence of more than 10,000 cases per million are located in the Americas, while a small number of countries in the eastern hemisphere have such a high prevalence. The variation in the COVID-19 mortality rate is much wider than the disease prevalence in the western and eastern hemispheres. The vast of the countries with death rates of more than 200 death per million are located in the western hemisphere including the Americas and Western European countries while such a high mortality rate has rarely occurred in the countries of the eastern hemisphere (Figure 2).

The geographical distribution of this disease does not seem to be related to the economic level of countries. If this was the case, it would be expected that the prevalence of disease and death rates due to COVID-19 would follow the pattern of the economic level of countries. Accordingly, the prevalence of the disease and mortality rates would be lower in high-income countries than in low-income countries. However, the geographical distribution of the disease does not indicate such a thing. Something that draws attention at first glance is that the geographical distribution of the disease varies widely in the west and east of the planet. It follows a kind of eastern and western distribution. Therefore, this variation seems to be beyond the economic level of countries.

Another issue that seems very strange about the geographical distribution of COVID-19 coronavirus is that the prevalence and death rate of the disease is very low at the origin of this disease. COVID-19 coronavirus was first detected in China and spread from there to other parts of the world. However, this country, which is the origin of the disease and the most populous country in the world, has been much less affected by the disease than many other countries. This mysterious geographical distribution in COVID-19 cases and deaths is a question that should be answered.

Besides the mysterious geographical distribution of COVID-19, many other aspects of this disease are still unknown and many unanswered questions remained. Where did COVID-19 coronavirus come from? Is the virus the result of human manipulation in a top-secret military lab or a natural mutation? Why this disease has had little effect on the country of origin as well as the countries of the eastern hemisphere, but it severely affected the Americas and Western Europe? Why does it spare some and kill others? Has the virus mutated during the pandemic period or may it mutate in the future and make the vaccine ineffective? We still have a long way to go to fully understand the disease and learn more about its mysterious characteristics.
